# Supplementary material for: DIRAS1 Drives Oxaliplatin Resistance in Colorectal Cancer via PHB1-Mediated Mitochondrial Homeostasis
Source: Biology (Basel). 2025 Jul 5;14(7):819. doi: 10.3390/biology14070819 (PMC12292402; doi:10.3390/biology14070819)
Supplement: Supplementary file 1 [file biology-14-00819-s001.zip › biology-3689011-supplementary.pdf]

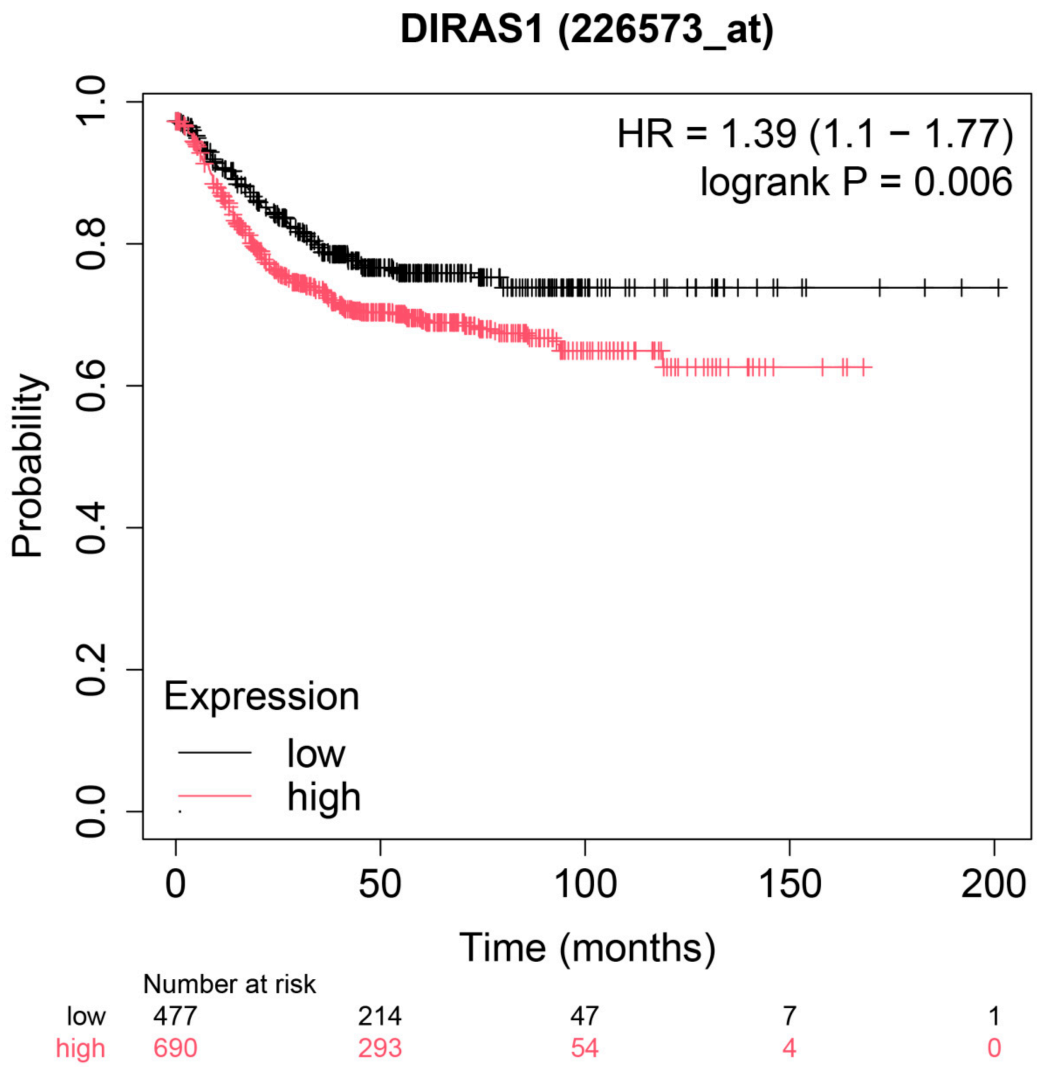

Figure S1 High expression of DIRAS1 was associated with the poor prognosis of patients with CRC

https://kmplot.com/analysis/index.php?p=service

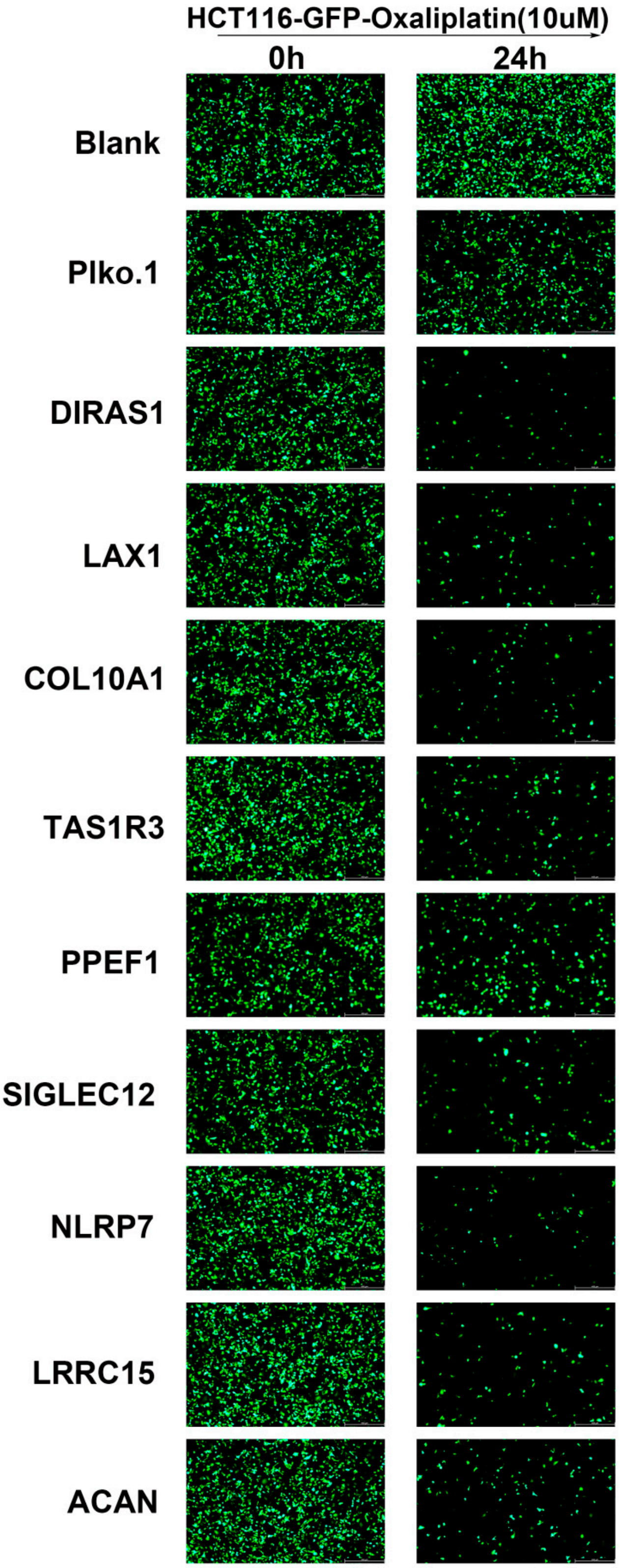

Figure S2. Survival of EGFP-positive cells was quantified by counting live cells with green fluorescence.

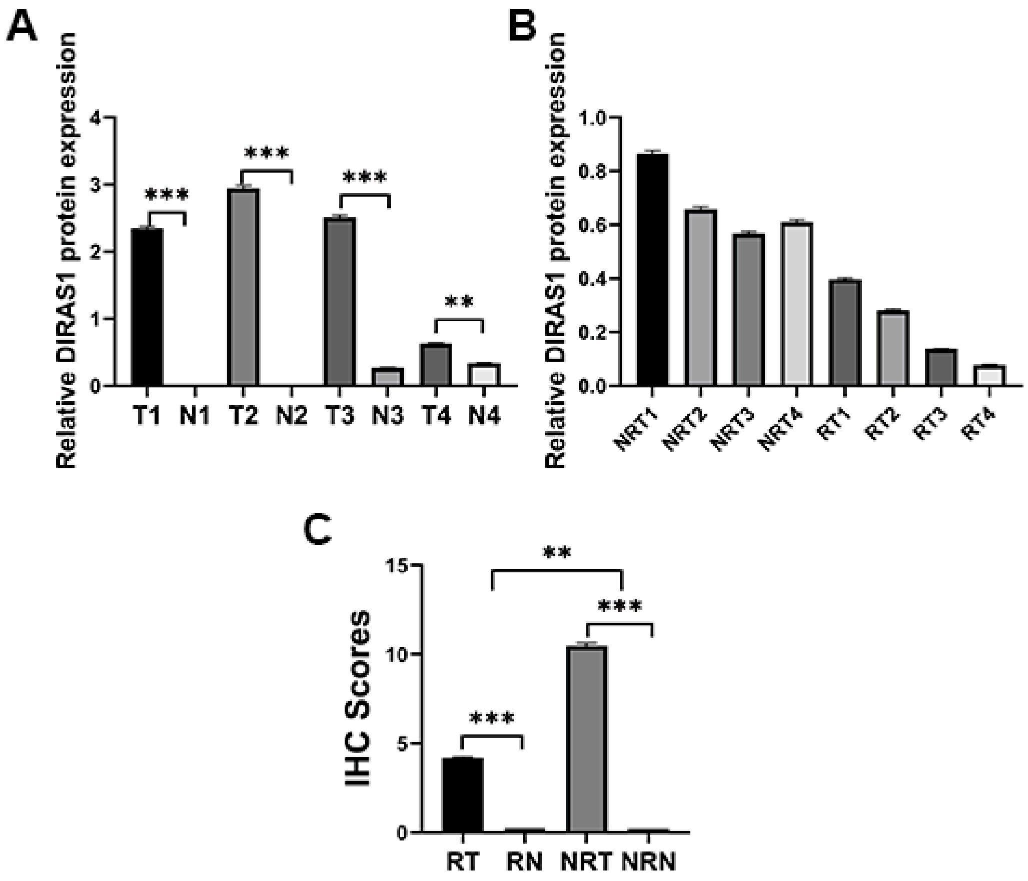

Figure S3.

(A,B) Semiquantitative analysis was used for protein quantification and ImageJ was utilized to analyze the gray value of protein bands.

(C). Immunohistochemical analysis of tissue and immunohistochemistry scores.

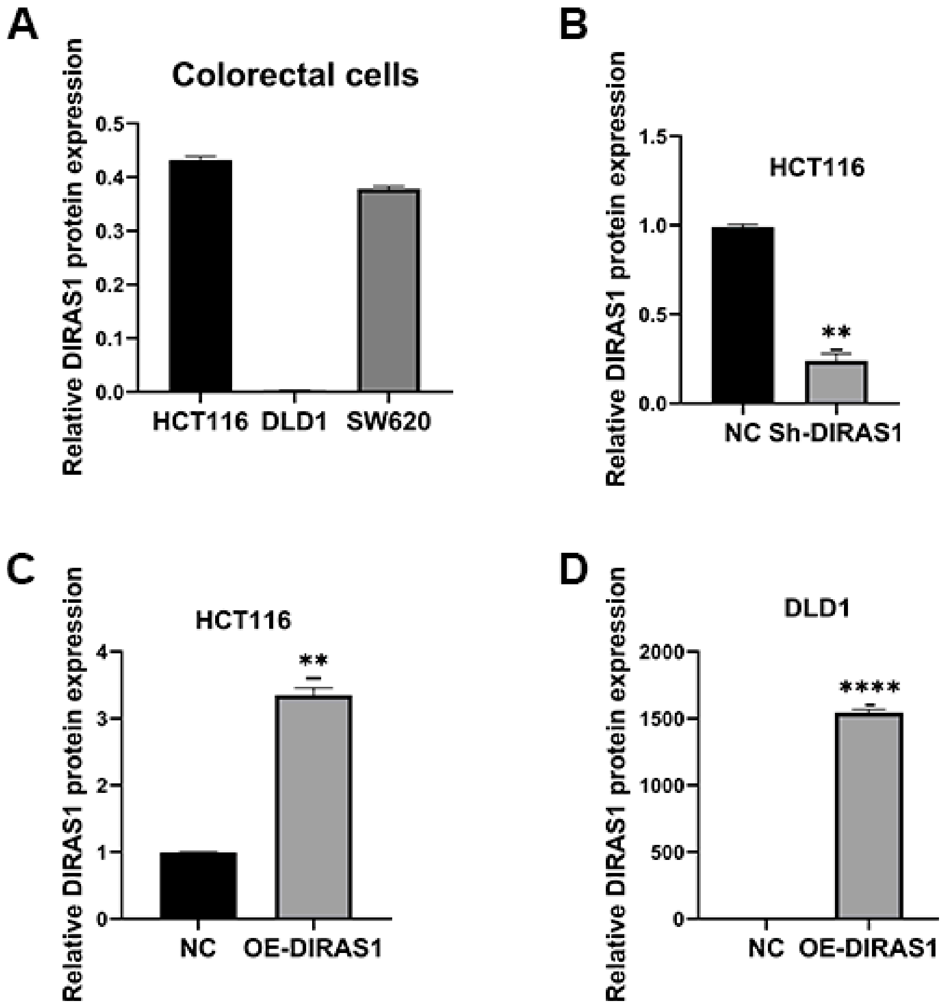

Figure S4. Semiquantitative analysis was used for protein quantification and ImageJ was utilized to analyze the gray value of protein bands.

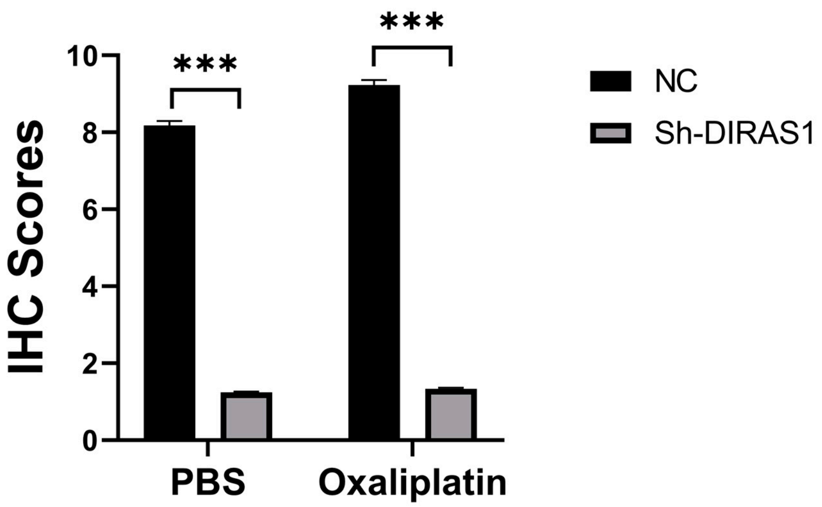

Figure S5. Immunohistochemical analysis of tissue and immunohistochemistry scores.

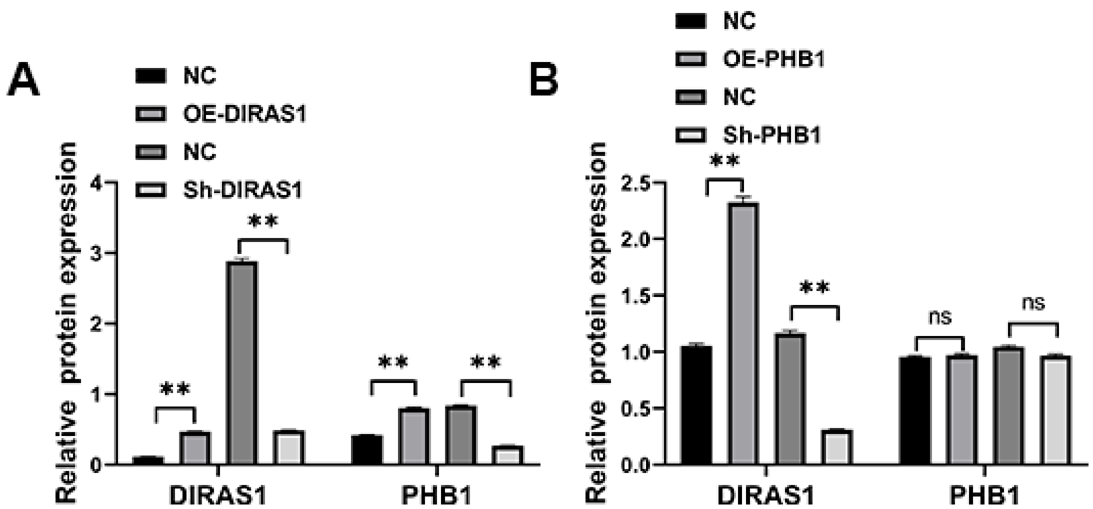

Figure S6. (A,B) Semiquantitative analysis was used for protein quantification and ImageJ was utilized to analyze the gray value of protein bands.

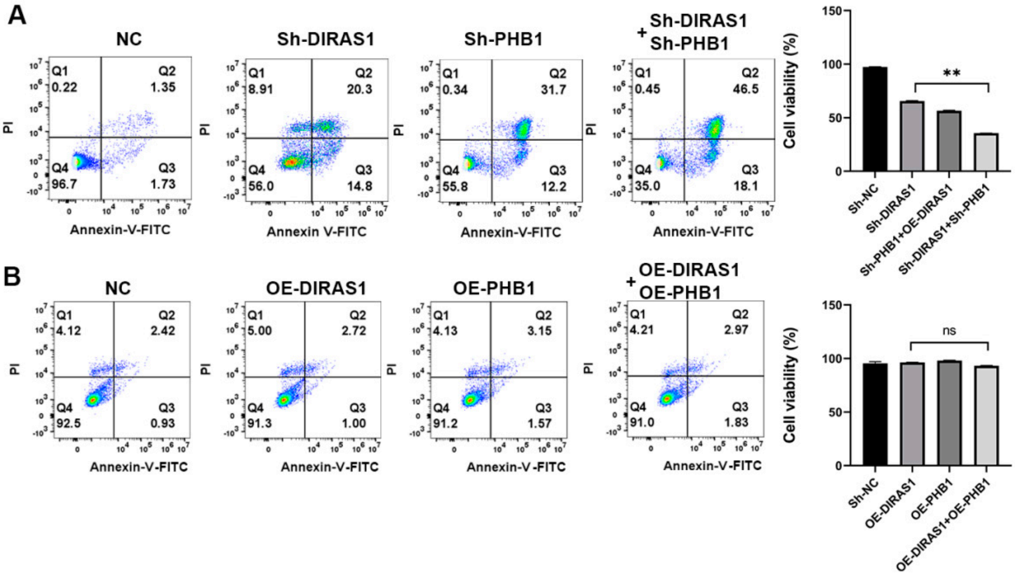

Figure S7. PHB1 is a Downstream Effector of DIRAS1.

(A) Flow cytometric analysis (Annexin V/PI staining) of late apoptosis (Annexin V^{+}/PI^{+}) in HCT116 cells after 48h treatment with 10 μM oxaliplatin. Groups:1.control (empty vector); 2. Sh-DIRAS1 (DIRAS1 shRNA + empty vector); 3.Sh-PHB1 (PHB1 shRNA + empty vector); 4. Sh-DIRAS1 + Sh-PHB1. Quantification (right): Proportion of viable (non-apoptotic, Annexin V^{-}/PI^{-}) cells (mean ± SD, n=3). Statistical significance (unpaired t-test): **p<0.01.

(B) Flow cytometry (setup as A). Groups:1. control(empty vector); 2. OE-DIRAS1 (empty vector + DIRAS1 expression vector); 3. OE-PHB1 (empty vector + PHB1 expression vector); 4. OE-PHB1 + OE-DIRAS1. Quantification (right): Proportion of viable cells (mean ± SD, n=3). Statistical significance (unpaired t-test): ns.

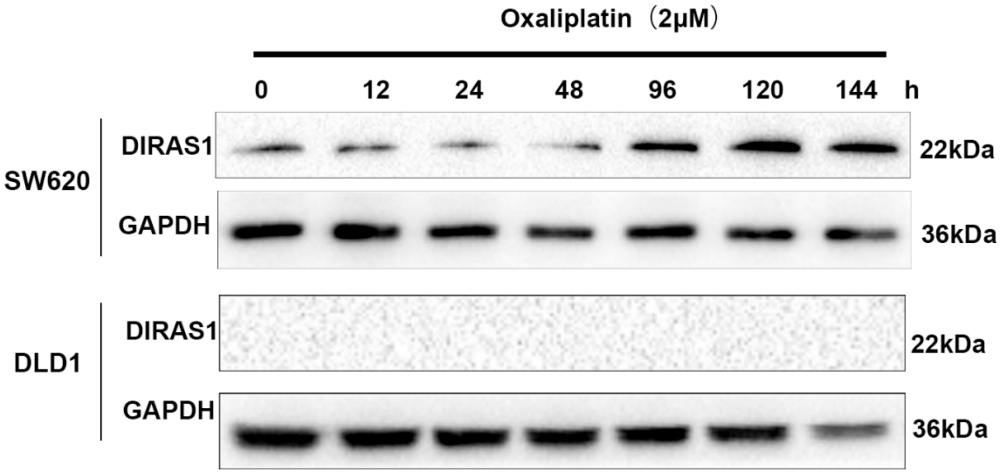

Figure S8 DIRAS1 expression is induced by OXA in a time-dependent manner. Western blot analysis of DIRAS1 expression in SW620/DLD1 cells treated with 2 μM OXA for indicated durations (0–144 h). GAPDH served as a loading control.

Table S1 Association of DIRAS1 expression with clinicopathologic features in colorectal tumor

Clinical factor	DIRAS1 expression level		*P* value
	Low	High	
Gender			
Male	12	14	0.7775
Female	13	11	
Age(year)			
>50	19	15	0.3635
≤50	6	10	
Tumor size(cm)			
>5	7	12	0.2436
≤5	18	13	
Differentiation			
Well	8	0	0.0085
Moderate	12	18	
Poor	5	7	
Tumor invasion			
T1	3	1	0.0239
T2	10	2	
T3	8	13	
T4	4	9	
Lymph node metastasis			
N0	21	7	0.0001
N1	4	18	
TNM stage			
I-II	19	7	0.0007
III-IV	6	18	

Chi-square test; *P < 0.05

Table S2 The expression profiles of DEGs

#ID	Symbol	CP_Count	CC_Count	CP_FPKM	CC_FPKM	FDR	log2FC	regulated
ENSG00000176490	DIRAS1	8	1034	0.242136	10.322668	6.34E-90	7.475844095	up
ENSG00000123500	COL10A1	32	2680	0.248797	29.089749	3.53E-109	6.869267169	up
ENSG00000254521	SIGLEC12	3	164	0.03689	2.798917	5.05E-35	6.192738049	up
ENSG00000086717	PPEF1	2	110	0.018987	1.298205	3.47E-26	6.168878052	up
ENSG00000169962	TAS1R3	16	375	0.12503	3.729295	9.85E-47	5.025806443	up
ENSG00000157766	ACAN	9	211	0.026107	0.837503	6.72E-35	5.016438572	up
ENSG00000172061	LRRC15	152	3302	0.705807	19.185841	2.12E-79	4.927616042	up
ENSG00000122188	LAX1	25	525	0.219275	5.930931	1.78E-51	4.871964585	up
ENSG00000167634	NLRP7	5	76	0.035064	0.729766	5.42E-16	4.374227535	up

Table S3 Five-year Outcomes

Months	DIRAS1-High	DIRAS1-Low
60	0	
60	0	
60		0
60		0
60		0
60		0
48		1
60	0	
60		0
60		0
60	0	
60	0	
60		0
60		0
60		0
60		0
60		0
60		0
48		1
45		1
40		1
39		1
27	1	
26	1	
25		1
24	1	
22	1	
21		1
21		1
20	1	
17	1	
16		1
16	1	
15	1	
15	1	
15		1
14	1	
14		1
14	1	
13	1	
12		1

12	1	
11	1	
11	1	
12		1
11	1	
10	1	
10	1	
9	1	
6	1	

0: Negative events

1: Positive events

Table S4 Differential expression analysis identified PHB1 as significantly upregulated in OE-DIRAS1 cells

#ID	Symbol	DIRAS1-OE	NC	OE/NC	log2FoldChange
ENSG00000187079	DIRAS1	1855	32	57.96875	5.857203472
ENSG00000186020	PHB1	506	64	7.90625	2.982993575
ENSG00000136603	HSP60	1392	187	7.443850267	2.896049036
ENSG00000058673	COX1	3086	476	6.483193277	2.696704583
ENSG00000131845	ATP1A1	189	40	4.725	2.240314329
ENSG00000157404	KIT	258	57	4.526315789	2.178337241
ENSG00000099219	ERMP1	770	174	4.425287356	2.14577114
ENSG00000176490	ATP5A	52581	12344	4.259640311	2.090731613
ENSG00000156875	COX5B	3331	785	4.243312102	2.085190795
ENSG00000100867	DHRS2	405	100	4.05	2.017921908
ENSG00000164327	RICTOR	1079	284	3.799295775	1.92573203
ENSG00000111186	WNT5B	172	46	3.739130435	1.902702799
ENSG00000163848	ZNF148	1518	409	3.711491443	1.891999043
ENSG00000130303	BST2	148	42	3.523809524	1.817135943
ENSG00000213928	IRF9	123	35	3.514285714	1.813231488
ENSG00000080493	SLC4A4	131	43	3.046511628	1.607158247
ENSG00000075420	FNDC3B	883	305	2.895081967	1.533604195
ENSG00000175662	TOM1L2	759	267	2.842696629	1.507260144
ENSG00000113140	SPARC	159	57	2.789473684	1.479992941
ENSG0000013	MATN2	107	39	2.743589	1.456064768

2561				744	
ENSG00000176788	BASP1	232	86	2.697674419	1.431716241
ENSG00000259522	AL136295.4	2171	835	2.6	1.378511623
ENSG00000139278	GLIPR1	88	34	2.588235294	1.371968777
ENSG00000176046	NUPR1	226	90	2.511111111	1.328325866
ENSG00000284874	AC000093.1	688	282	2.439716312	1.286713402
ENSG00000168918	INPP5D	173	71	2.436619718	1.284881108
ENSG00000026036	RTEL1-TNFRSF6B	134	55	2.436363636	1.284729477
ENSG00000280987	RTEL1-TNFRSF6B	13417	5539	2.42227839	1.276364682
ENSG00000139269	INHBE	135	56	2.410714286	1.269460675
ENSG00000285816	AP000944.2	519	218	2.380733945	1.251406404
ENSG00000168702	LRP1B	121	51	2.37254902	1.246437896
ENSG00000169752	NRG4	212	90	2.355555556	1.236067359
ENSG00000103740	ACSBG1	127	54	2.351851852	1.233797185
ENSG00000256043	CTSO	192	82	2.341463415	1.227410496
ENSG00000156466	GDF6	78	34	2.294117647	1.197939378
ENSG00000160207	HSF2BP	121	53	2.283018868	1.190942783
ENSG00000164307	ERAP1	614	274	2.240875912	1.164062762
ENSG00000270800	RPS10-NUDT3	685	306	2.238562092	1.162572335
ENSG00000213760	ATP6V1G2	131	59	2.220338983	1.150779952
ENSG00000077092	RARB	214	97	2.206185567	1.141554144
ENSG00000180190	TDRP	189	86	2.197674419	1.13597767
ENSG0000016	ST6GALNA	1315	603	2.180762	1.124832892

0408	C6			852	
ENSG00000125384	PTGER2	76	35	2.171428571	1.118644496
ENSG00000185633	NDUFA4L2	71	33	2.151515152	1.105353
ENSG00000284773	AC114490.3	66	31	2.129032258	1.090197809
ENSG00000143217	NECTIN4	80	38	2.105263158	1.074000582
ENSG00000131095	GFAP	128	61	2.098360656	1.069262663
ENSG00000204390	HSPA1L	96	46	2.086956522	1.061400545
ENSG00000048052	HDAC9	87	42	2.071428571	1.050626073
ENSG00000213967	ZNF726	92	45	2.044444444	1.031708859
ENSG00000270181	BIVM-ERCC5	1043	515	2.025242718	1.01809482
ENSG00000175697	GPR156	87	43	2.023255814	1.016678741
ENSG00000157502	MUM1L1	107	53	2.018867925	1.013546532
ENSG00000264343	NOTCH2NLA	180	90	2	1
ENSG00000159885	ZNF222	122	61	2	1
